# Management of an Aorto-Hepatic Artery Conduit Pseudoaneurysm and Stricture in a Patient After a Liver Transplant

**DOI:** 10.7759/cureus.77720

**Published:** 2025-01-20

**Authors:** Timothy M Cooke, Christopher Harnain, Benjamin May

**Affiliations:** 1 Interventional Radiology, NewYork-Presbyterian Weill Cornell Medical Center, New York, USA; 2 Interventional Radiology, State University of New York Downstate College of Medicine, Brooklyn, USA

**Keywords:** aorto-hepatic artery conduit, arterial rupture, arterial stent, interventional cardiology, interventional radiology, post liver transplant, pseudo aneurysm

## Abstract

Aorto-hepatic artery conduits (AHC) are created in patients with unsuitable native hepatic arteries after a liver transplant, usually from donor internal iliac arteries. There is scarce literature describing the management of vascular complications, such as pseudoaneurysms (PSAs) and strictures, in a tenuous AHC. We present the case of a 37-year-old female liver transplant recipient who was found to have an expanding AHC PSA and anastomotic stricture. Using interventional radiology equipment, we restored the flow to the patient’s liver graft while managing an intra-procedural rupture of the PSA. A working knowledge of the equipment used by interventional cardiologists in the interventional radiology suite can prove invaluable for managing complex arterial interventions in patients with liver transplants.

## Introduction

An extra-hepatic hepatic artery pseudoaneurysm (HAPA) is a rare but well-documented complication that can develop after liver transplantation with an incidence of 2-3% and reported mortality rates up to 80% [1,2]. A HAPA usually forms in patients with risk factors such as primary sclerosing cholangitis (PSC), Roux-en-Y hepaticojejunostomy, and bile leaks [3]. In liver transplant patients whose native hepatic arteries are already unsuitable for use due to factors such as hepatic artery thrombosis, inability to dissect the hepatic artery, or unusual anatomy, aorto-hepatic conduits (AHC) are created to provide arterial inflow to the transplanted liver [4,5]. Although there are multiple reported cases describing HAPA complications and their management [6], to our knowledge, there are no previous cases in the literature describing the management of a conduit hepatic artery pseudoaneurysm (CHAPA).

Here, we describe our unique management of a young woman with a CHAPA and conduit anastomotic stricture, including the application of interventional angioplasty and stenting devices for this complex anatomy. 

## Case presentation

A 37-year-old female patient underwent a deceased donor liver transplant and Roux-en-Y anastomosis for the treatment of her PSC and cholangiocarcinoma. Two months following the liver transplant, the patient underwent a celiac artery ligation, distal pancreatectomy, splenectomy, and formation of an AHC due to an expanding retroperitoneal hematoma secondary to the pancreatic leak. The pancreatic leak was likely due to an iatrogenic injury from the three separate endoscopic retrograde cholangiopancreatographies (ERCP) and incomplete healing due to prior radiation therapy. The AHC was created using a donor internal iliac artery and originated from the infrarenal aorta. It joined the patient’s native common hepatic artery with an end-to-side anastomosis, using 5-0 polypropylene sutures. A computed tomography angiogram (CTA) was completed after the surgery to demonstrate patency of the conduit and the hepatic arteries.

Diagnostic Angiogram

**Figure 1:**
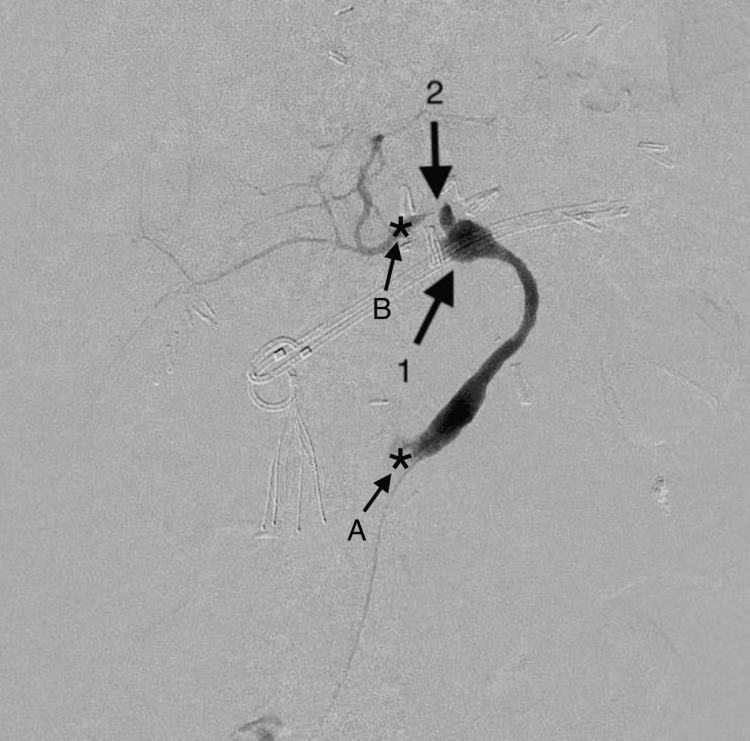
Digital subtraction angiogram of the pseudoaneurysm (1) and severe anastomotic stricture (2) of the aorto-hepatic conduit in anterior-posterior view. The asterisks indicate the anastomosis of the conduit with the aorta (A) and the native hepatic artery (B)

Seven months after the AHC formation, the patient presented to the emergency department with complaints of abdominal pain and palpitations. A CT abdomen and pelvis demonstrated concern for a CHAPA. A CTA confirmed an arterially enhancing outpouching measuring 1.6 x 1.1 cm and a severe stricture of the conduit immediately distal to the pseudoaneurysm (PSA; Figure 1).

The patient was deemed a poor surgical candidate due to ongoing inflammation from a chronic pancreatic leak. A plan was made to re-establish arterial flow to the liver by stenting the stricture with cardiac drug-eluting stents (DES) and performing stent-assisted coiling of the CHAPA.

Intervention

Working from the right groin, a 5 French (Fr) sheath was advanced to the origin of the AHC. A 5 Fr angled catheter (Cook Medical, Indiana, United States) was advanced proximal to the aneurysm. The patient was heparinized with 75 mg/kg unfractionated heparin, and the aneurysm and stricture were crossed using a standard 016 Fathom wire (Boston Scientific, Massachusetts, United States) and a 2.4 Progreat catheter (Terumo Medical Corporation, New Jersey, United States).

The system was then exchanged for interventional cardiac devices to enable stenting of this complex and tortuous anatomy. An 014 Hi-Torque Wiggle guide wire (Abbott Laboratories, Illinois, United States) was advanced into place to enable tracking of the DES. Over this wire, a 4.0 x 32 mm Synergy stent (Boston Scientific, Massachusetts, United States) was deployed across the stricture through a 5 Fr Ansel sheath and over-dilated to 5.0 mm. A second 4.0 x 48 mm Synergy stent was extended across the PSA and over-dilated to 5.75 mm.

**Figure 2:**
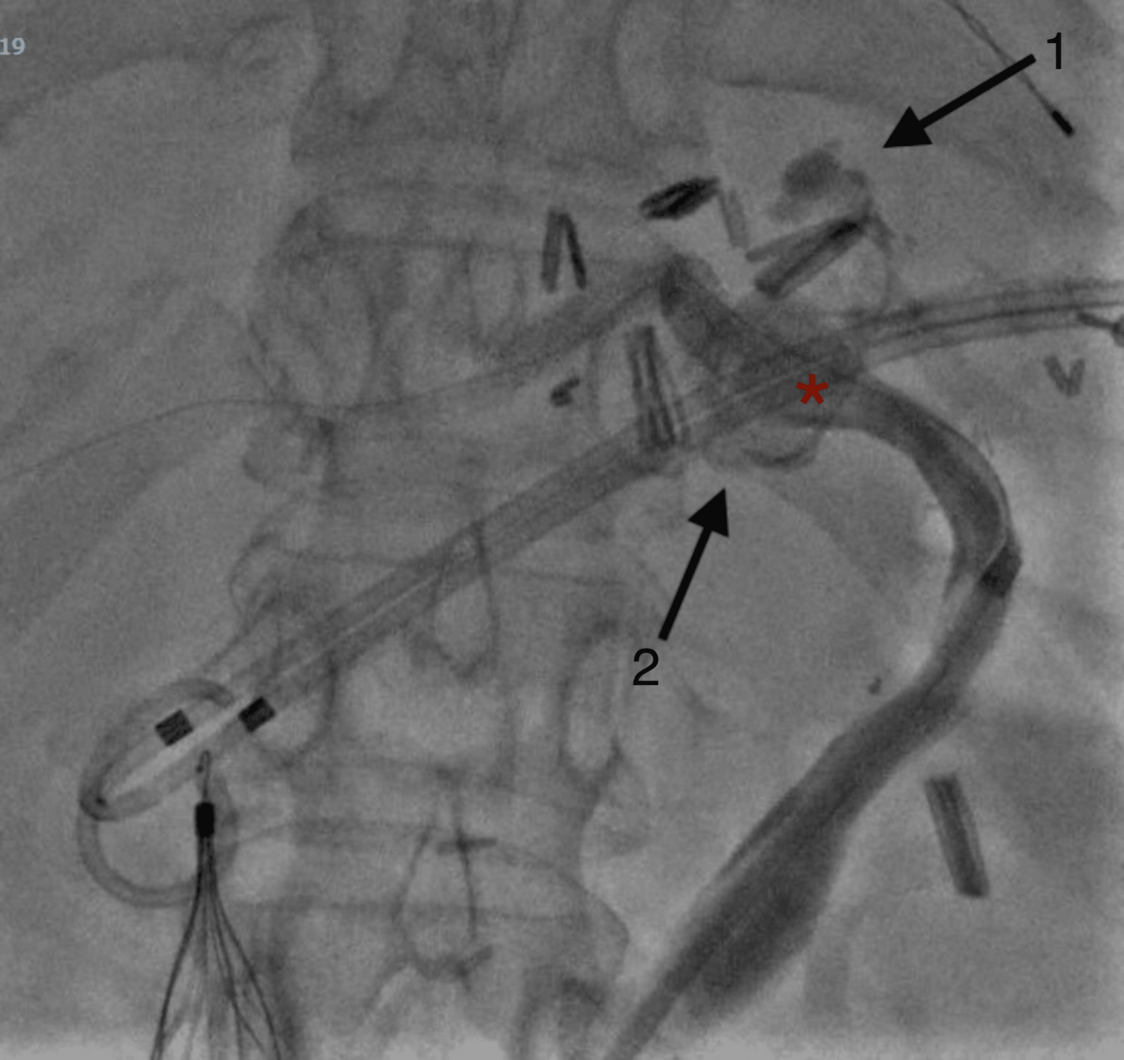
Rupture of aorto-hepatic conduit artery with contrast extravasation demonstrated superior (1) and inferior (2) to previous site of pseudoaneurysm (red asterisk).

However, a contrast injection demonstrated a rupture of the CHAPA, compromising all arterial blood flow to the liver and eliminating the possibility of stent-assisted coiling (Figure 2).

**Figure 3:**
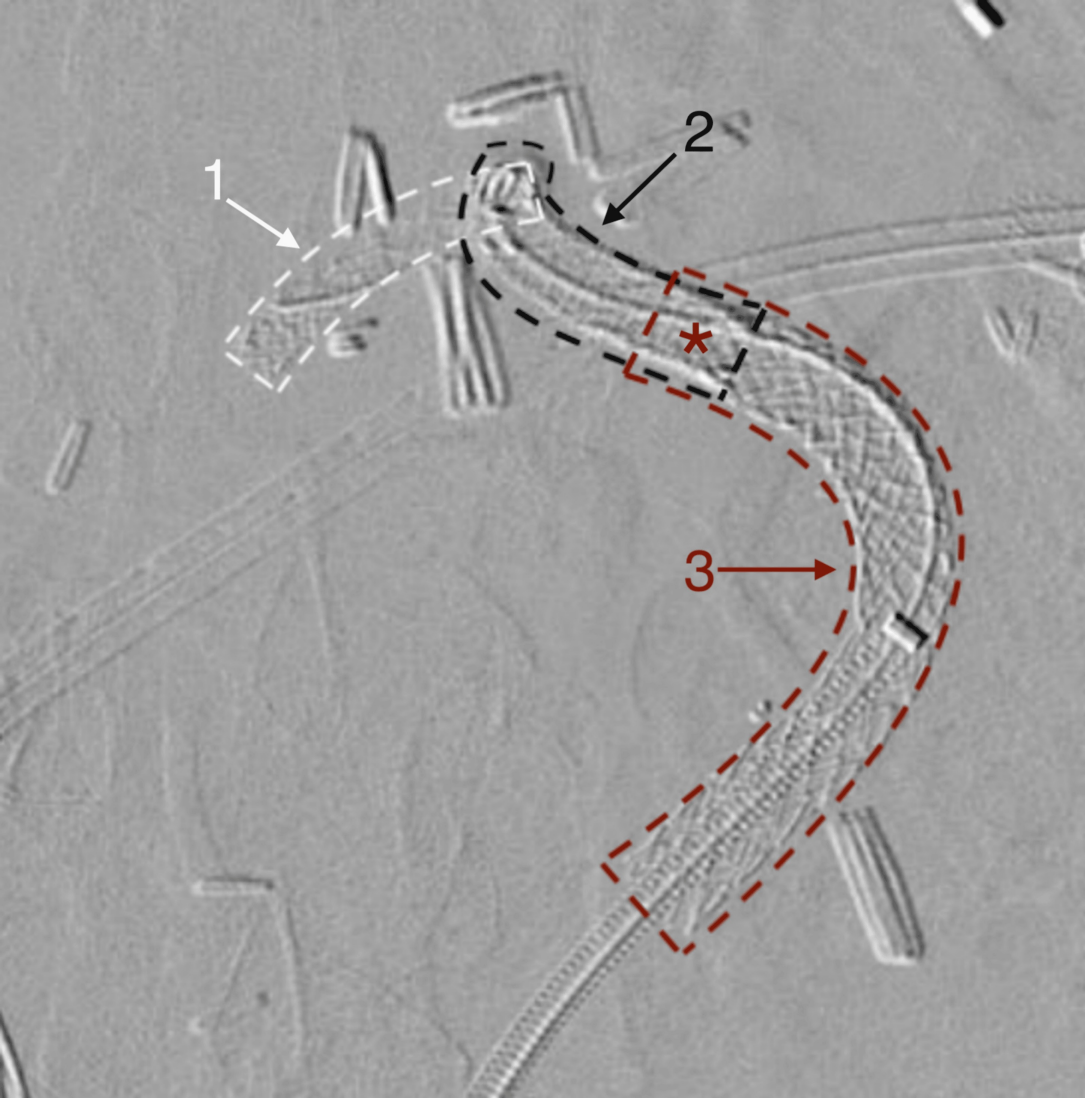
Digital subtraction angiogram demonstrating the location of the three stents The first Synergy stent (1) extends across the end-to-side anastomosis and stricture. The second Synergy stent (2) overlaps with the first Synergy stent and extends across the stricture and pseudoaneurysm (red asterisk). The GORE VIABAHN VBX covered stent (3) overlaps with the second Synergy stent to cover the ruptured pseudoaneurysm.

**Figure 4:**
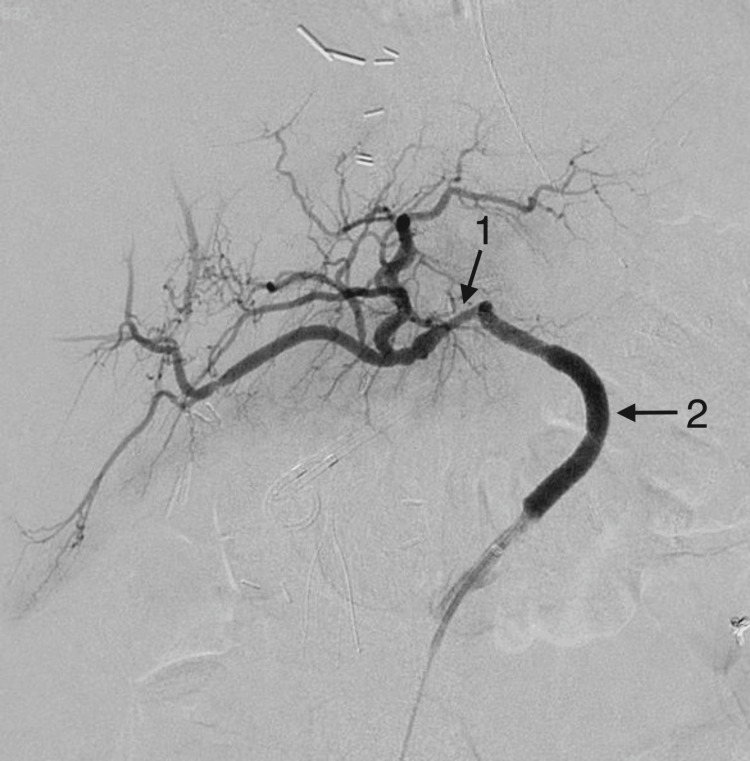
The final digital subtraction angiogram after placement of the two 4.0 mm Synergy stents (1) and a 7.0 mm GORE® VIABAHN® VBX covered stent (2)

The groin access was emergently upsized to a 7 Fr sheath for the placement of a 7.0 mm x 8 cm GORE VIABAHN VBX (Gore Medical, Delaware, United States) covered stent which was over-dilated to 8.0 mm. This stent was deployed over an 035 Glidewire Advantage wire (Terumo Medical Corporation, New Jersey, United States). The final digital subtraction angiograms demonstrated complete exclusion of the ruptured CHAPA and brisk flow to the patient’s liver graft (Figures 3, 4).

The patient recovered without complication and with normal graft function. She was discharged on Plavix (clopidogrel) 75 mg daily and therapeutic Eliquis (apixaban) 2.5 mg twice per day. The hemoglobin and hematocrit levels were monitored during the patient's five-day postoperative hospital stay and once a week for the first month on anticoagulation. Eliquis was stopped after one month due to heavy menstrual bleeding. Follow-up CTA at one month and three months have confirmed the patency of the system.

## Discussion

The Pittsburgh group first described AHCs as alternative methods of arterialization of the hepatic graft nearly 35 years ago [7]. Vascular complications of AHCs, such as CHAPAs, are extremely rare due to the fact that so few patients require an AHC, and there is scarce data regarding the rate of vascular complications that develop in AHCs. In transplant patients, a conduit vessel is only created when the native hepatic artery becomes unsuitable for use [4,5]. Although the reported 10% occlusion rate for AHCs is significantly higher than the 4% occlusion rate of vessels in non-conduit transplant cases [5], data regarding the management of the vascular complications associated with such a tenuous vessel, such as thrombosis [8] and the PSA and stricture, as in our patient, are virtually nonexistent. Although Doppler ultrasound has been considered the initial diagnostic tool of choice to detect vascular complications after liver transplantation [9], information on endovascular techniques and strategies for managing complex AHC interventions in such patients is sparse.

Because PSA presentations and complications can be so variable [10,11], especially in an AHC, it was important to employ novel techniques to solve this unique problem. In this case, specialized interventional cardiac equipment designed for tortuous cardiac anatomy proved quite useful in managing complex arterial intervention. Firstly, the 014 Hi-Torque Wiggle guide wire has a stainless-steel core and sinusoidal shape that provides excellent back support for tracking stents into place. The stiffer nature of the wire allowed for quick navigation through the PSA and stenosis despite the tortuosity and rapid stent deployment. Secondly, just like the guide wire, the balloon-mounted drug-eluting Synergy stents tracked extremely well through the patient’s tortuous anatomy. The non-compliant cardiac balloons can also be sized at smaller 0.25 mm increments, thus preventing overdilation and allowing for finite adjustments to the stent diameter. Lastly, the sinusoidal shape of the system allowed additional stents and catheters to track through the deployed stents without engaging the interstices.

Despite these advantages, the CHAPA ruptured intra-procedurally. However, due to the extremely tenuous nature of the AHC, various sizes of covered stents were already available in the interventional radiology suite, with surgery on standby in case the patient required further intervention. It was difficult to predict how the AHC would respond to these interventions, so it was important to be prepared for any scenario, including rupture of the CHAPA. Having these measures in place while intervening on the AHC allowed for the quick sealing of the rupture.

## Conclusions

Managing vascular complications such as PSAs, strictures, and rupture in AHCs can be quite challenging. It is rare for a patient to require an AHC, but failure to manage conduit complications can lead to life-threatening consequences. A working knowledge of the equipment used by interventional cardiology can prove invaluable for these difficult cases. Additionally, anticipating failure points, especially the rupture of the CHAPA, is essential to quickly seal the rupture.

In conclusion, minimally invasive management of CHAPAs after liver transplantation can be augmented with a comprehensive understanding of interventional tools and techniques. However, prospective studies comparing surgical revascularization and endovascular intervention are needed to determine optimal management while considering the hemodynamic status of the patient. Future studies should also investigate the potential impact of conduit type and prophylactic anti-aggregation on arterial patency and graft survival.
